# Comparison of mMO-TLIF via Midline Incision Versus MIS-TLIF via Wiltse Approach in Lumbar Degenerative Disease

**DOI:** 10.1007/s43465-024-01150-2

**Published:** 2024-05-15

**Authors:** Shengwen Li, Zhiqiang Zhou, Fanjian Meng, Xinyu Huang, Maohua Cheng, Yixin Shen, Peng Zhang, Zhengfeng Lu, Qianghua Zeng

**Affiliations:** 1 Department of Orthopedics, Haining People’s Hospital, Jiaxing, 314400 China; 2 Department of Orthopedics, The Second Affiliated Hospital of Soochow University, Suzhou, 215004 China; 3 Department of Orthopedics, Suzhou Hospital of Integrated Traditional Chinese and Western Medicine, Suzhou, 215101 China; 4 Suzhou Medical College, Soochow University, Suzhou, 215004 China

**Keywords:** Lumbar degenerative disease, Transforaminal lumbar interbody fusion, Mini-open, Minimally invasive

## Abstract

**Background:**

To compare the clinical and radiological outcomes of modified mini-open transforaminal lumbar interbody fusion (mMO-TLIF) via posterior midline incision for "targeted limited dissection" versus minimal invasive transforaminal lumbar interbody fusion (MIS-TLIF) via Wiltse approach in lumbar degenerative diseases.

**Methods:**

A total of 60 consecutive patients in our center from January 2019 to March 2020 were enrolled, including 30 patients who were treated with mMO-TLIF via posterior midline incision and 30 treated with MIS-TLIF through the Wiltse approach. Perioperative parameters were recorded. The questionnaires of Oswestry Disability Index (ODI) and Visual Analogue Score (VAS) were conducted before the operation and after the operation (3 days, 1 week, and 2 years). CT and MRI radiological outcomes were evaluated before the operation and at a 2-year follow-up.

**Results:**

There were no significant differences in the general data, gender, age, and BMI between the two groups. All patients were successfully operated without intraoperative complications. There were significant differences between the two groups in the operation time (*p* < 0.001) and intraoperative bleeding (*p* < 0.05). There was no difference in ODI and VAS scores between groups pre- and post-operatively, but they were both significantly improved compared to those before the operation (*p* < 0.01). At a 2-year follow-up, the paraspinal muscle atrophy and fat infiltration were increased comparing to pre-operation, but the difference was also not statistically significant (*p* > 0.05). In addition, both the two groups’ fusion rates were more than 90% at a 2-year follow-up, however, no difference was detected between the two groups.

**Conclusion:**

mMO-TLIF via midline incision for “targeted limited dissection” could achieve similar clinical and radiological outcomes as MIS-TLIF for lumbar degenerative disease.

## Introduction

Transforaminal lumbar interbody fusion (TLIF) is a conventional operation for treating lumbar spinal stenosis. The conventional-open TLIF (CO-TLIF) requires extensive bilateral dissection of muscle fibers attached to the vertebral lamina using the middle incision, a major trauma to the paravertebral tissue structure. In addition, the long-time traction of paraspinal muscles and the secondary ischemia and denervation during the operation often leads to long-term postoperative back pain, and even failed back surgery syndrome (FBSS) may occur [1].

With the introduction of the concept of minimally invasive spine surgery, a variety of minimally invasive TLIF (MIS-TLIF) and mini-open TLIF (MO-TLIF) have been explored and gained growing popularity [2–5]. In particular, Wiltse first proposed to operate in the intermuscular plane between the multifidus muscle and longissimus lumborum [6], and most of the later studies believed that the Wiltse approach had the advantages of less invasion of paraspinal muscle, less postoperative pain, faster recovery, shorter hospital stay and so on [7, 8]. However, in clinical practice, we find that the distance between the Wiltse plane and the posterior midline gradually increases due to the course and hypertrophy of multifidus muscle fibers in the lower lumbar spine, which makes the operation in the lower lumbar spine very inconvenient. In addition, to fully expose the visual field of TLIF through the Wiltse approach, it is necessary to use various types of retractor devices. Besides, prolonged muscle traction by the devices also leads to muscle ischemia and denervation [9].

**Fig. 1:**
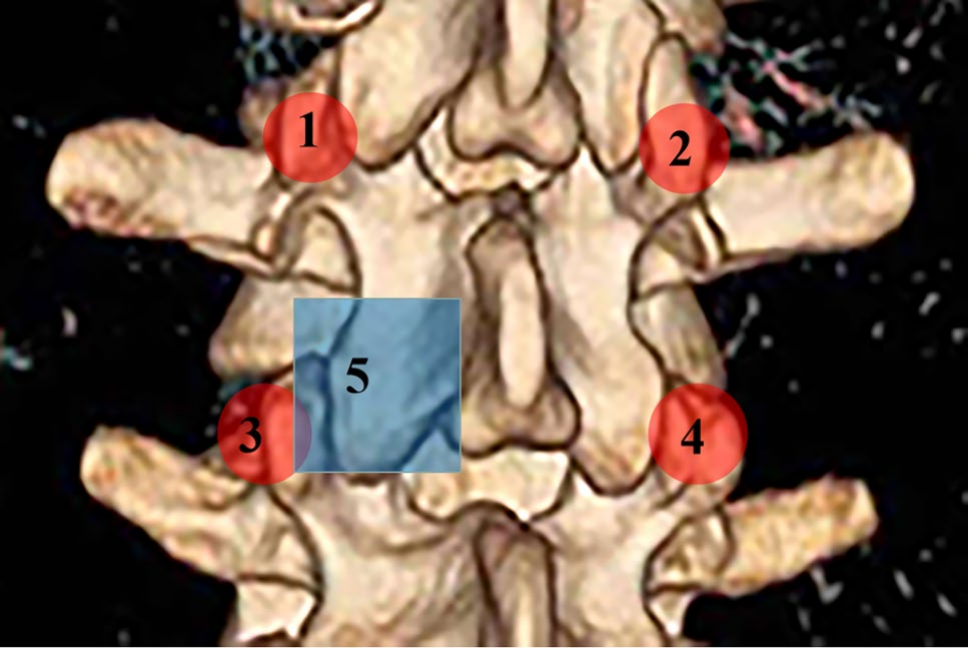
The procedure diagram shows four screw placement areas (1–4: Red-circle marking) and one laminectomy area (5: Blue-square marking). The operation procedure was described as “step-by-step” from the contralateral side to the symptomatic side and “targeted limited dissection” for “point exposure”

Given this, in recent years, many surgeons have tried to return to the posterior midline plane for spine surgery [10, 11]. In particular, the establishment of "the para-midline fatty plane" and "trans-multifidus approach" further verified the feasibility of MIS-TLIF or MO-TLIF via the posterior midline incision [11, 12]. On this basis, we improved the conventional subperiosteal dissection through posterior midline incision to the limited dissection of screw placement and laminectomy areas through the fatty plane, which avoided violent traction injury to paraspinal muscles, and the damage to spinal nerve branches and dorsal branches of lumbar artery (Fig. 1). In addition, the modification in surgical procedures for "step-by-step" from contralateral side to symptomatic side and "targeted limited dissection" for "point exposure" could also avoid the long-term continuous traction and invasion of paraspinal muscles. After clinical verification, the modified MO-TLIF (mMO-TLIF) could realize screw placement, laminectomy, and interbody fusion using a 4-5cm midline incision length without the assistance of special retractor equipment.

In the past two decades, minimally invasive spine surgery with the help of various retractors and endoscopy has been prevalent [13]. However, adhering to minimally invasive surgery and improving operation details and procedures based on conventional-open surgery is also a research topic. This study compares the clinical and radiological outcomes of mMO-TLIF without the special retractor via the posterior midline incision and MIS-TLIF with the Quadrant retractor via the Wiltse approach in lumbar degenerative diseases.

## Methods

### Study Design

This is a quasi randomized trial study, approved by the Institutional Review Board of the Second Affiliated Hospital of Soochow University. The single-level TLIF was performed for degenerative lumbar diseases, including lumbar disc herniation, lumbar spinal stenosis, and lumbar spondylolisthesis of grade I. The main indications were as follows: (1) MRI and CT showing obvious lumbar disk herniation with segmental instability confirmed by dynamic radiography; (2) MRI and CT showing lumbar foraminal stenosis with segmental instability confirmed by dynamic radiography; (3) Grade 1 lumbar spondylolisthesis with Intervertebral disc degenerative; (4) Failure of conservative treatment for 6 months, or progressive symptoms. Patients were excluded for the following reasons: (1) Lumbar spondylolisthesis grade 2 or above; (2) Neoplastic spondylopathy, spinal infections and fractures involving lumbar vertebrae; (3) Reoperation; (4) Serious co-morbidities, such as diabetes, hypertension with poor control and respiratory infection.

According to the above criteria, 60 (33 females and 27 males) out of 98 patients with single-level degenerative lumbar diseases were recruited in this study from January 2019 to March 2020. The patients were randomly divided into two groups: mMO-TLIF group (30 patients) and MIS-TLIF group (30 patients). Thirty-four patients were diagnosed with lumbar disk herniation with segmental instability, 12 with lumbar spinal stenosis with segmental instability and 14 with lumbar spondylolisthesis. There are no special retractor tools used in the mMO-TLIF group via the posterior midline incision; while the Quadrant tubular retractor was applied in the MIS-TLIF group via Wiltse posterolateral. All patients completed follow-ups at 3 days, 1 week and 2 years postoperatively. CT scans and MRI were performed to evaluate the fusion status, paraspinal muscle area, and degree of fat infiltration at a 2-year follow-up.

### Surgical Procedure

**Fig. 2:**
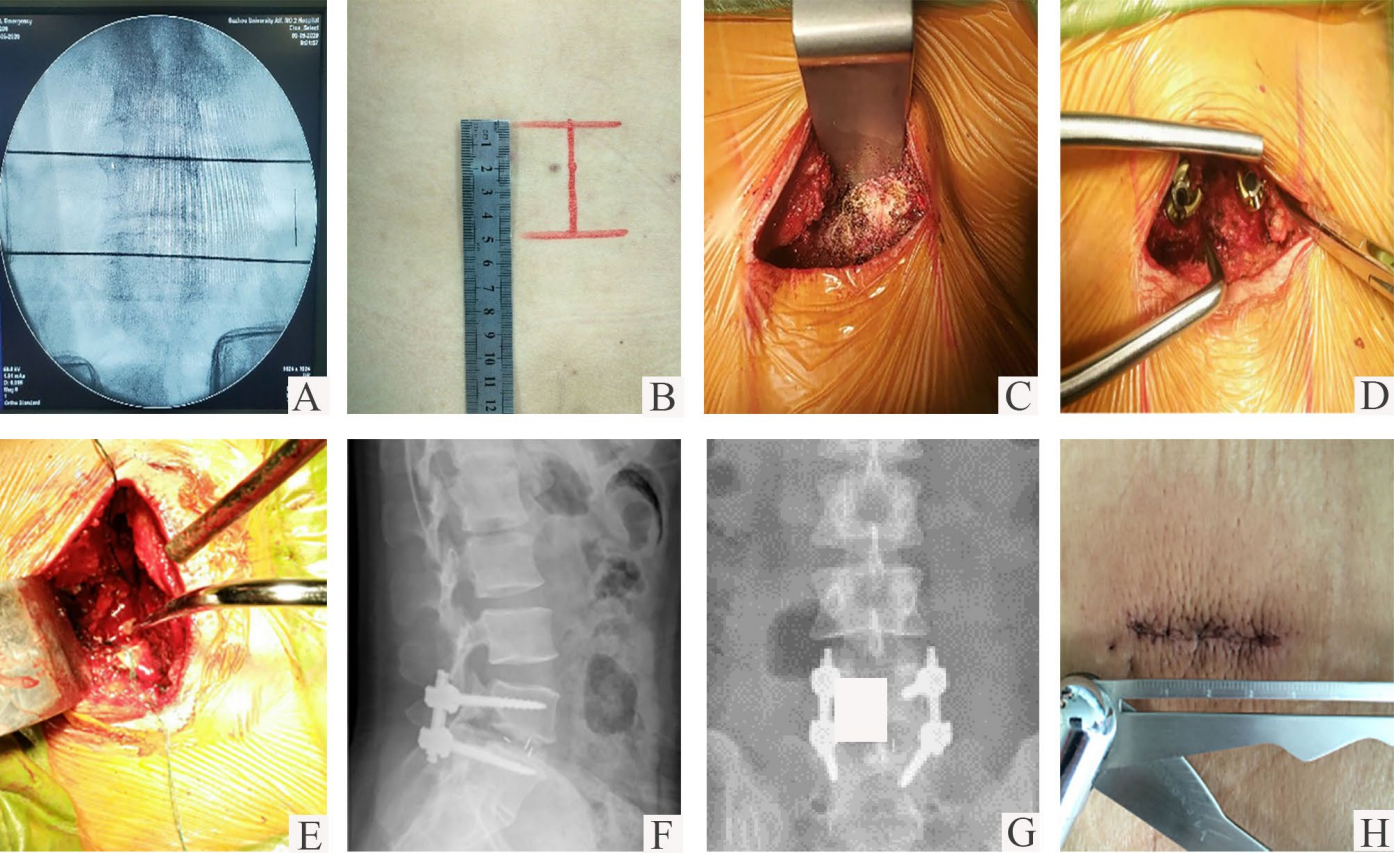
Surgical procedure of mMo-TLIF (modified Mini-Open Transforaminal Lumbar Interbody Fusion) via posterior midline incision. Preoperative localization of the surgical segment (**a**) and marked skin incisions (**b**); Along the Para-midline fat plane "point exposed" the pedicle screw entry area (**c**) and placement of the screw one by one (**d**); The "point exposure" for laminectomy with conventional lamina retractor (**e**); The anteroposterior and lateral X-ray results after operation (**f**, **g**); The appearance of midline 4.0 cm incision length (**h**)

In the mMO-TLIF group, the essence of the operation is as follows: A 4–5 cm posterior midline incision is initiated, carefully made over the spinous processes of the targeted vertebral levels. Following the skin incision, the deep lumbar fascia is meticulously exposed through the subcutaneous fat layer. Cerebellar retractors are strategically employed to gently retract the skin and subcutaneous tissues, ensuring minimal tissue disturbance. Deviating from the traditional approach, the deep lumbar fascia is not incised directly in the midline. Instead, a longitudinal incision is made bilaterally adjacent to the spinous processes, maintaining a safe distance of approximately 5 mm from these structures and deep to the fascia. This meticulous dissection allows for the transection of the multifidus muscles, revealing the para-midline fatty plane. Focused dissection on the hemilamina of the symptomatic side is then pursued within this fatty tissue plane, carefully staying just medial to the deep paraspinal muscles. The conventional periosteal stripper and lamina retractor are adeptly utilized to achieve point exposure, revealing the facet capsule. This capsule is then exposed by gently releasing the surrounding musculature, taking care to preserve the integrity of the capsule itself. With the facet capsules exposed at all desired levels, they can subsequently be removed as necessary to facilitate decompression and fusion procedures. The screw placement, subtotal facetectomy, discectomy, and interbody fusion are skillfully executed from the contralateral side to the symptomatic side, as depicted in Fig. 2.

**Fig. 3:**
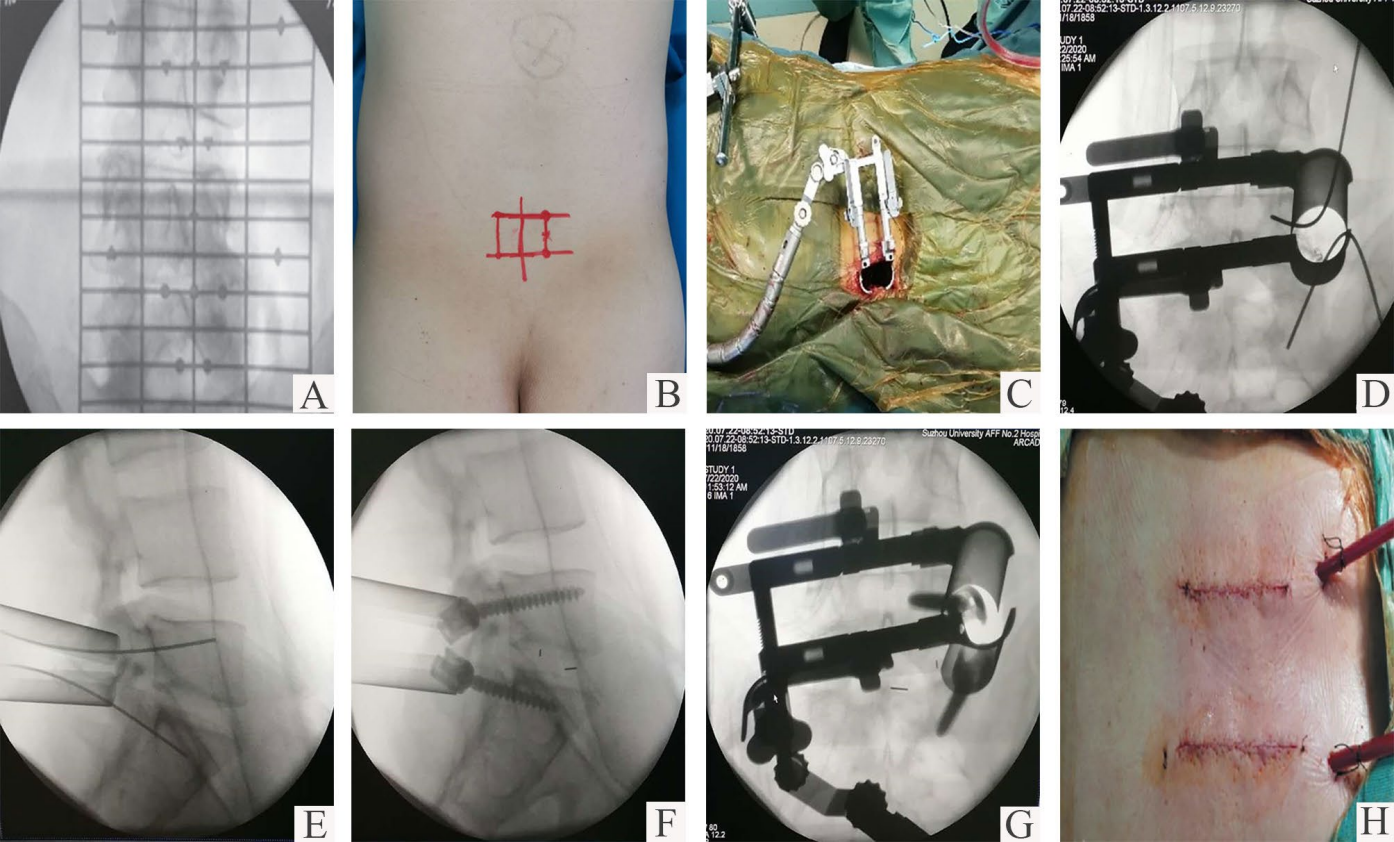
Surgical procedure of MIS-TLIF (minimal invasive transforaminal lumbar interbody fusion) via Wiltse approach. Preoperative localization of the surgical segment (**a**) and marked skin incisions (**b**); The Quadrant^®^ expansion tube established the working channel through the Wiltse approach (**c**); The anteroposterior and lateral intraoperative imaging of Guide Wire insertion via transpedicular access (**d**, **e**) and Implanted pedicle screws (**f**, **g**); The appearance of bilateral 3 cm incision length (**h**)

In the MIS-TLIF group, the procedure is as follows: Initial incision localization is expertly guided by C-arm fluoroscopy, ensuring pinpoint accuracy. Subsequently, carefully mark the incision at a distance of approximately 2.5 cm from the midline on the symptomatic side, with an appropriate length of 2.5–3.5 cm for each incision. The skin and fascia are then incised with care, followed by the strategic insertion of the Quadrant^®^ expansion tube (Medtronic Sofamor Danec, Memphis, TN). The subsequent stages of screw insertion, subtotal facetectomy, discectomy, and the critical interbody fusion are seamlessly conducted by the same senior surgeons, ensuring the highest standard of surgical excellence. This meticulous approach is encapsulated in Fig. 3.

All pedicle screws used were LEGACY^®^ (Medtronic Sofamor Danec, Memphis, TN), and interbody fusion cage CAPSTONE^®^ (Medtronic Sofamor Danec, Memphis, TN) was selected.

### Assessment of Results

The observation indicators included the questionnaire of Oswestry Disability Index (ODI) and Visual Analogue Score (VAS) was conducted before operation and after operation (3 days, 1 week, and 2 years). Perioperative parameters were recorded on time, including operation time, intraoperative bleeding, the error rate of pedicle screw placement, postoperative drainage volume, hospital stay, incision length, and degree of swelling around the incision. CT was used to evaluate the interbody fusion rate at a 2-year follow-up, and MRI (Siemens, Germany) was used to evaluate the changes in bilateral paraspinal muscle area and fat infiltration before the operation and at a 2-year follow-up. The multifidus and erector spine area was measured in the MRI T2-weighted axial images of the horizontal plane of intervertebral disc space to avoid artifacts caused by implants. The total/lean cross-sectional area and the ratio of fat infiltration for multifidus and erector spinae before surgery and 2 years after surgery were measured by the software (Scion Corp, Frederick, MD).

The degree of swelling includes I–III degrees. Grade I means slight swelling of the skin and the skin texture still exists; Grade II means that the swelling is more obvious, and the skin texture disappears, but there were no blisters; Grade III means that the swelling is pronounced, and the blisters appear. Moreover, the scores are 3, 2, and 1 [14].

Fusion rates were assessed by CT scanning with 3-mm slices at a 2-year follow-up using the Brantigan-Steffee-Fraser (BSF) classification [15]. A classification of BSF-3 was considered as fusion status.

The degree of fat infiltration includes 0 to III degrees, 0 degrees without fiber and fat infiltration; degree I, fiber and adipose tissue area < 10%; II degree, the area of the fiber and adipose tissue is 10–50%; III degree, the area of the fiber and adipose tissue is > 50%. Furthermore, it is recorded as 3, 2, 1, and 0, respectively [16].

### Statistical Analysis

All continuous data were presented as mean ± standard deviation (SD), and categorical data were presented as percentage or number ratio. For the univariate comparative analysis, unpaired t-test or non-parametric Kruskal–Wallis test was used to compare continuous data between groups, and the chi-square test was used to compare ratios between groups. All data were analyzed using SPSS 20.0 (IBM Corp., Armonk, NY, USA), and a *p* value less than 0.05 was considered statistically significant.

## Results

### Demographic Data and Perioperative Morbidity

There were no significant differences in the general data, gender, age, and BMI between the two groups. There was no significant difference in the diagnosis constituent ratio between the two groups (*p* = 0.451), and in the fused segment between the two groups (*p* = 0.938) (Table 1). According to postoperative CT imaging evaluation, 3 pedicle screws were penetrating the lateral cortical bone of the pedicle in the mMO-TLIF group. In comparison, 4 screws penetrated the lateral cortex, and 1 screw penetrated the medial cortex of the pedicle in the MIS-TLIF group. Neither group had serious complications such as nerve root, spinal cord, and vascular injuries. In the mMO TLIF group. In addition, there was one case of postoperative local skin necrosis, the use of local debridement and continuous dressing changes resulted in delayed wound healing.

**Table 1:** Demographic data for the patients in the two groups

		mMO-TLIF	MIS-TLIF	*P*
	Gender (female/male)	17/13	16/14	0.795^*^
	Age (years)	63 (60.25,68.75)^†^	62.5 (59.25,68.5)^†^	0.594^※^
	BMI	23.57 ± 2.14	23.67 ± 2.20	0.859^※^
Etiology			*χ*^2^ = 1.594	0.451
	Disc herniation	16	18	
	Lumbar spinal stenosis	5	7	–
	Lumbar spondylolisthesis	9	5	
Fusion levels			*χ*^2^ = 0.128	0.938^*^
	L3/4	6	5	
	L4/5	13	14	–
	L5/S1	11	11	–

^†^Values are expressed as median (a quarter, three quarters)

^※^*p*-value was calculated using the Mann-Whitney *U* test

^*^p-value was calculated using the Chi-square test

There were significant differences between the two groups in the operation time (mMO-TLIF: 105.83 ± 6.86 min vs. MIS-TLIF: 116.47 ± 8.19 min; *p* < 0.001) and intraoperative bleeding (mMO-TLIF: 149.70 ± 14.17mL vs. MIS-TLIF: 156.13 ± 7.28mL; *p* = 0.043). There was no significant difference in postoperative blood loss, hospital stay and degree of swelling between the two groups. At 3 days and 1-week post-surgery, the degree of swelling of the two groups was significantly reduced compared with that of 1 day after the operation. However, the swelling degree of the two groups at the same time point was not statistically significant (Table 2).

**Table 2:** Comparison of clinical outcomes between the two groups in the perioperative period

		mMO-TLIF	MIS-TLIF	p^*^
Operation time (min)		105.83 ± 6.86	116.47 ± 8.19	<0.001
Intra-operative blood loss (ml)		149.70 ± 14.17	156.13 ± 7.28	0.032
The length of surgical incision (cm)		5.00 ± 0.46	6.24 ± 0.22	<0.001
Post-operative blood loss (ml)		196.80 ± 9.30	199.03 ± 10.90	0.396
Hospital stay (days)		7.27 ± 1.14	7.47 ± 1.11	0.494
Degree of swelling	1 day after operation	2.43 ± 0.63	2.47 ± 0.63	0.838
	3 days after operation	2.63 ± 0.61	2.67 ± 0.55	0.825
	1 week after operation	2.800 ± 0.47	2.90 ± 0.31	0.286

Note: Values are expressed as mean ± standard deviation

^*^p-value was calculated using the paired *T*-test

### Clinical Outcomes

**Fig. 4:**
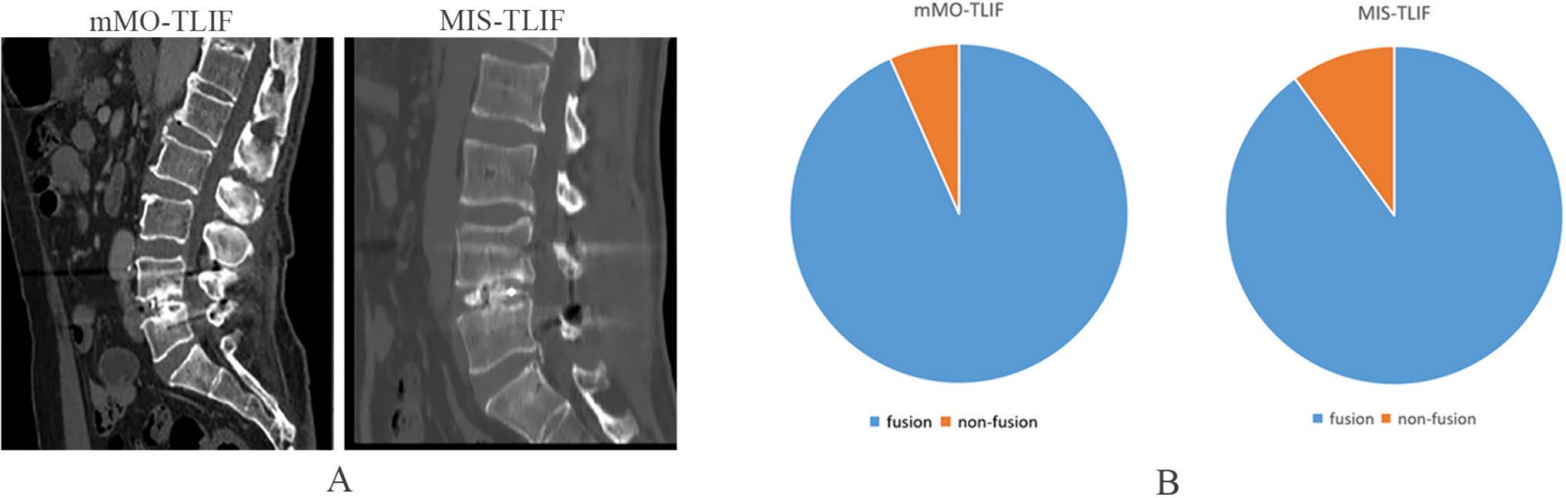
Comparision of ODI scores between preoperation and the 2-year follow-up between the mMO-TLIF and the MIS-TLIF groups (**a**); Comparision of VAS scores at preoperation, postoperative 3 days, 1 week, and 2-year follow-up in both groups (**b**)

There were no differences between the two groups in ODI and VAS scores before the operation (*p* > 0.05). The ODI and VAS scores decreased significantly with the extension of follow-up time. However, there was no significant difference in ODI score at 2-year follow-up and VAS score at postoperative 3 days, 1 week, and last follow-up between the two groups (*p* > 0.05) (Fig. 4). The comparison of clinical results before and after operation was statistically significant (*p* < 0.01) (Table 3).

**Table 3:** Comparison of ODI and VAS scores in the follow-up

		mMO-TLIF	MIS-TLIF	p^*^
ODI	Pre-operation	38.53 ± 3.71	39.57 ± 4.22	0.318
	3 days post-operation	23.40 ± 2.01	23.63 ± 2.24	0.792
	1 week post-operation	18.87 ± 2.75	18.07 ± 2.79	0.283
	2 years post-operation	15.93 ± 2.07	15.57 ± 2.05	0.493
VAS	Pre-operation	6.37 ± 1.19	6.43 ± 1.14	0.825
	3 days post-operation	3.27 ± 0.94	3.37 ± 0.81	0.661
	1 week post-operation	1.80 ± 0.61	1.87 ± 0.63	0.678
	2 years post-operation	1.40 ± ± 0.50	1.37 ± 0.49	0.795

Note: Values are expressed as mean ± standard deviation

^*^p-value was calculated using the paired *T*-test

### Imaging Results

**Fig. 5:**
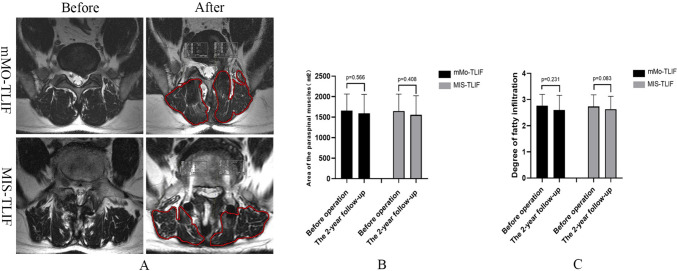
The CT scan showed the interbody fusion and bone-bridge formation in the two groups (**a**); a Comparision of fusion rate between the mMO-TLIF group (93.3%) and MIS-TLIF group (90%) (**b**)

**Fig. 6:**
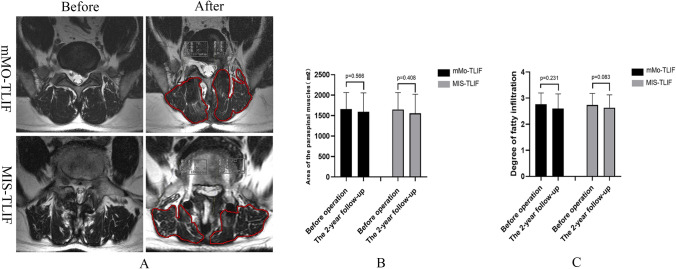
Typical MRI images for calculating the area of the paraspinal muscles and fatty infiltration degree evaluation between mMO-TLIF and MIS-TLIF groups before operation and 2-year follow-up (**a**). Comparison of the cross-sectional area (**b**) and the degree of fatty infiltration (**c**) in paraspinal muscles in both groups before operation and 2-year follow-up

According to BSF classification, 2 cases in the mMO-TLIF group did not achieve the complete fusion of interbody (Fusion rate: 93.3%). In comparison, 3 cases in the MIS-TLIF group were not fused (Fusion rate: 90%) (Fig. 5). The area of the paraspinal muscles and the degree of fatty infiltration had no significant changes in the two groups before and after the operation (*p* > 0.05). At the 2-year follow-up, MRI showed that the area of paraspinal muscles in the two groups was smaller than before the operation, and the degree of fat infiltration was more serious than before the operation. However, the difference was not statistically significant (*p* > 0.05). In addition, the area of paraspinal muscles measured in the mMO-TLIF group (pre-operation:1660.10 ± 402.93 mm^2^; The 2-year follow-up:1591.40 ± 464.72 mm^2^) was larger than that in the MIS-TLIF group (pre-operation:1648.27 ± 413.01 mm^2^; The 2-year follow-up:1555.30 ± 467.55 mm^2^), and the degree of fat infiltration in the mMO-TLIF group (pre-operation:2.77 ± 0.43; The 2-year follow-up:2.63 ± 0.49) was also smaller than that in the MIS-TLIF group (pre-operation:2.73 ± 0.45; The 2-year follow-up: 2.60 ± 0.56), but no significant difference was observed (*p* > 0.05) (Fig. 6).

## Discussion

The conventional posterior median approach of TLIF has a wide range of invasion to paraspinal muscles, which is prone to damage the posterior ramus of the spinal nerve located in the muscle and increase the intramuscular pressure. There is no doubt that these MIS-TLIF or MO-TLIF procedures have reported more advantages than CO-TLIF procedures in terms of the average blood loss, mean hospital stay, quality of life, and so on. Many literatures have proposed several elements of MIS-TLIF [13, 17]: (1)The use of some special retractors (including non-expandable and expandable), (2) Incision and approach are more conducive to screw placement and laminectomy decompression, (3) Proper use of various visualization aids such as surgical loupes, microscope or endoscope. In our MIS-TLIF procedure, we used a bilateral paramedian incision through the Wiltse approach with the aid of a Quadrant tubular retractor, which met the various elements of MIS-TLIF mentioned above.

With the wide development of MIS-TLIF, many deficiencies are emerging. The reliance on special retraction devices restricts the wide use of MIS-TLIF, especially in undeveloped countries and regions, and long-term use of retractors may also lead to muscle injury, ischemia, and denervation, and even lead to iatrogenic compartment syndrome[14, 18–20]. Because of this, some surgeons have proposed to turn back to the midline incision and modify the part of surgical procedures of CO-TLIF for mini-open spinal surgery. In our mMO-TLIF surgery, there are several elements included: (1) The surgical anatomy is modified from extensive subperiosteal dissection to “targeted limited dissection”. In addition to the symptomatic side of the laminectomy decompression area, the other areas only used Cobb periosteal stripper to perform blunt dissection of the fatty plane, reduce the use of electrotome and avoid the injury of the posterior ramus of the spinal nerve. (2) Adhere to the principle of micro invasion for "point exposure" during operation without special retractor equipment. (3) The operation procedure was described as a "step-by-step" process in four screw placement areas and one laminectomy decompression area. First, the contralateral screws were placed, and then the symptomatic side screws and decompression were performed.

Currently, there are growing researches about MIS-TLIF compared with CO-TLIF in the treatment of lumbar degenerative diseases. Most of these studies pointed to the obvious advantages of MIS-TLIF operation in terms of intraoperative bleeding and hospital stay. However, there was no significant difference in operation time and blood loss in most works of literature, even worse than that of CO-TLIF [21–24]. Coincidentally, the mMO-TLIF operation based on the CO-TLIF performed better than MIS-TLIF in terms of operation time and blood loss in our study. Moreover, mMO-TLIF surgery also overcomes the disadvantages of conventional surgery in terms of intraoperative bleeding and hospital stay. Although we did not set the CO-TLIF group as the control group, the perioperative parameters of the mMO-TLIF still showed a significant improvement advantage. The reason may be related to the improvement of the CO-TLIF technique and procedure. In the previous literature, the degree of incision swelling and incision length were rarely reported. Our study shows that the first 3 days after the operation is a serious period of swelling and dehydration, and detumescence drugs should be used properly. In addition, the incision for mMO-TLIF surgery is completed by a midline incision, which is relatively simple and easy to operate. Moreover, the length of the incision in mMO-TLIF surgery has a significant advantage over the total length of the double incisions in MIS-TLIF surgery.

ODI and VAS scores as the most commonly used evaluation indicators in most literatures. The existing studies always showed that the scores of MIS-TLIF and CO-TLIF are significantly improved after the operation. However, when it comes to the comparison between groups at different time points, there is great divergence [23]. This is partly consistent with our current research results. Both mMO-TLIF and MIS-TLIF could significantly improve the clinical efficacy of the patients. However, there were no significant statistical differences in ODI and VAS scores between groups at the same time point. From a statistical point of view, we believe that mMO-TLIF has the same advantages as MIS-TLIF compared with CO-TLIF.

The previous literature evaluated the fusion rate between vertebrae by CT or X-ray [24]. Here, we choose CT as the evaluation standard. The fusion rate of the two groups at a 2-year follow-up was more than 90%, which coincided with other studies [25, 26]. In our research, the autogenous bone fragments made from decompressed lamina bone tissue are enough to provide ideal bone fusion without the need for additional BMP, allogeneic, or artificial bone. In addition, MRI is often used to estimate paraspinal muscle atrophy and fat infiltration, but there are few reports about MRI evaluation before and after TLIF. Luis Alberto Ortega-Porcayo et al. [27] retrospectively analyzed the MRI data of 11 patients who underwent unilateral MIS-TLIF and unilateral pedicle screw placement. By comparing the functional cross-sectional area of multifidus and erector spinalis on the operative and non-operative sides, it was found that MIS-TLIF through a mini-open tubular approach produced minimal paraspinal muscle damage. Wu et al*.* [28] evaluated the edema and atrophy of multifidus muscle with T2 weighted magnetic resonance imaging (MRI) at 3 different time points (preoperative, postoperative, and 1-year follow-up). They reported the safety and efficiency of a novel inextensible endoscopic tube for TLIF. Tian et al. [29] compared a modified MIS-TLIF and TLIF through the Wiltse approach with MRI score and atrophy rate of CSA and verified the advantages of the new modified operation. Our MRI results showed no significant difference in the paraspinal muscle atrophy and degree of fat infiltration between the preoperative and postoperative groups. This indicates that the mMO-TLIF has the advantages of MIS-TLIF, which can invade paraspinal muscle less.

This study has some limitations. First of all, there was no CO-TLIF operation as a control group. Although there is a lot of existing literature on the comparative study of MIS-TLIF and CO-TLIF, mMO-TLIF is a new operation method, and there is still a lack of randomized controlled parameters with CO-TLIF. Secondly, the sample size is small and follow-up time are relatively short. Undoubtedly, our results need to be further verified by long-term large sample size with randomized controlled trials. In addition, all operations were performed by senior surgeons in the single medical center. Therefore, there is a lack of universality of technical capability, which may lead to partial data bias.

## Conclusions

The mMO-TLIF via the posterior midline incision for "targeted limited dissection" and MIS-TLIF via the Wiltse approach achieve similar clinical and radiological satisfaction for lumbar degenerative disease. The mMO-TLIF is beneficial to tissue exposure, operation time, and intraoperative bleeding.

## Data Availability

All data generated during this study are included in this published article and its supplementary information files.

## Abbreviations

CO-TLIF: Conventional-open TLIF
mMO-TLIF: Modified mini-open transforaminal lumbar interbody fusion
MIS-TLIF: Minimal invasive transforaminal lumbar interbody fusion
ODI: Oswestry Disability Index
TLIF: Transforaminal lumbar interbody fusion
VAS: Visual Analogue Score
